# Specificity of homicide in tunisian women

**DOI:** 10.1192/j.eurpsy.2023.1873

**Published:** 2023-07-19

**Authors:** R. Ouali, N. Smaoui, A. Chamseddine, I. Gassara, R. Feki, M. Maalej, N. Charfi, J. Ben Thabet, L. Zouari, S. Omri, M. Maalej

**Affiliations:** psychiatry C department, Hedi Chaker University Hospital, sfax, Tunisia

## Abstract

**Introduction:**

Homicide is the most serious, radical and irreversible criminal act. It arouses a multitude of questions and fears. Committed by women, the homicide seems more unusual and weird.

**Objectives:**

Our objective is to describe the circumstances of the homicide committed by Tunisian women

**Methods:**

This study was retrospective and descriptive. It focused on the files of criminal psychiatric expertise and involved female accused subjects.We have collected all the criminal expert reports carried out over a period of 24 years (from January 1, 1998 to December 31, 2021)

**Results:**

This study included 21 women who committed homicide.The majority of victims of homicide or attempted homicide (85.6%) belonged to the family circle of the accused.Five women (23.8% ) committed this act against their children and four women (19.1%) committed this act against their husbands.The means most used in homicide and its attempt were immolation (23.8%), strangulation (23.8%) and blows (23.8%).

Among those charged with homicide or its attempt, seven (33.3%) presented a personality disorder, 14.3% a depressive disorder, 14.3% a moderate intellectual disability, 4.8% a schizophrenia and 4. 8% mild intellectual disability.

**Conclusions:**

This female criminality, in particular homicide, would find their explanations in different factors and the existence of a mental disorder at the origin of deviant behavior in women seems to be a significant factor in the determinism of such behavior. Much effort remains to be made to apply preventive measures.

**Disclosure of Interest:**

None Declared

